# To be understood: Transitioning to adult life for people with Autism Spectrum Disorder

**DOI:** 10.1371/journal.pone.0194758

**Published:** 2018-03-26

**Authors:** Craig Thompson, Sven Bölte, Torbjörn Falkmer, Sonya Girdler

**Affiliations:** 1 Cooperative Research Centre for Living with Autism (Autism CRC), Long Pocket, Brisbane, Queensland, Australia; 2 School of Occupational Therapy, Social Work and Speech Pathology, Curtin University, Perth, Western Australia, Australia; 3 Department of Women’s and Children’s Health, Pediatric Neuropsychiatry Unit, Center of Neurodevelopmental Disorders at Karolinska Institutet (KIND), Child and Adolescent Psychiatry Research Center, Karolinska Institutet, Stockholm, Sweden; 4 Child and Adolescent Psychiatry, Center for Psychiatry Research, Stockholm County Council, Stockholm, Sweden; IRCCS E. Medea, ITALY

## Abstract

**Introduction:**

The purpose of this study was to explore the viewpoints of parents of young people with Autism Spectrum Disorder (ASD) in relation to their child’s transition to adulthood.

**Methods:**

Data were collected during four structured focus groups with 19 parents of young people with ASD with average to high intellectual capacities. Condensed meaning units were identified and checked during focus groups, and were subsequently linked to the International Classification of Functioning, Disability and Health (ICF).

**Results:**

Three major themes emerged: to be understood, to understand the world and to succeed. The ICF domains of activity and participation and environmental factors emerged as having the greatest potential to influence transition outcomes.

**Conclusions:**

Policies and services should focus on strengths to maximise participation in higher education, employment and independent living amongst young people with ASD. Interventions targeting environmental factors could be effective in improving participation in adult life. Person-centred and individualised approaches could further complement this approach supporting the transition to adulthood for people with ASD, ultimately improving outcomes in adulthood.

## Introduction

Leaving school and transitioning to adulthood presents challenges for all adolescents and their families, however this can be particularly stressful period for families living with disabilities including Autism Spectrum Disorder (ASD) [1]. The estimated prevalence of ASD has been gradually increasing and is now thought to be approximately 1% across the lifespan [2, 3]. This trend is likely to continue as evidenced by the Australian context, where 75% of people diagnosed with ASD are 19 years old or younger [4]. While young people with ASD are ambitious and aspirational [5], they commonly experience poor transition outcomes including unemployment [6], limited participation in further education [7] and low rates of independent living [8], with most having few friendships [9, 10].

Poor vocational and educational outcomes are sustained across the lifespan for those with ASD with average or above average intellectual capacities [11], outcomes resulting at least in part from the limited appropriate and affordable services available to this population as they transition into adulthood [5]. Current services are constrained by funding limitations and professionals appropriately trained to work with adults with ASD [11, 12]; having been criticised for lacking coherence and evidence to support the efficacy of their interventions [13, 14]. This service environment results in many young people with ASD and their families experiencing high levels of unmet needs [5], and struggling to navigate the transition process largely by themselves [15].

Education is recognised as a basic human right [16]; in Australia equity in education has been supported by the Disability Discrimination Act (DDA) [17]. The DDA [17] has a strong focus on promotion of equity through social inclusion, and has positively impacted the education of people with disabilities[18] evidenced by the 94% increase in students with disabilities attending university since 2008 [19]. However, the DDA [17] does not mandate transition planning, a recognised gap in the current system [20], with many young people with disabilities still struggling to find clear pathways during their transition to adulthood.

Families are an integral support to their young person with ASD as they transition to adulthood [21]. Parents continue to be a major source of support and advocacy for young people with ASD even as they move into adult life [22], and focuss primarily on securing an acceptable future for their young person’s life [23]. The value of familial support in improving transition outcomes is evident in its positive impact on employment post-school [24], and in the role that parental expectations play in mediating the relationship between leaving school and outcomes in adulthood [25]. However, the finding that parental expectations for transition outcomes are lower for parents of children with ASD than for parents of children with intellectual disability or multiple disabilities is cause for concern [26].

Person-centred approaches have been suggested as a means of addressing the needs of the young person with ASD during the transition to adulthood [27]. However, the development of such approaches are constrained by a lack of knowledge and understanding of the experiences, hopes and wishes of parents of young people with ASD as they transition to adulthood. This is problematic, as support services decrease as young people with ASD move into adult life [28]. The overall aim of this study was to explore the experience of parents during the transition to adulthood for young adults with ASD. Specific research objectives included identifying factors that supported the transition process and unmet needs during this period in Australia.

## Method

### Design

An inductive approach was utilised to gain an understanding of the experience of families of young adults with ASD in normative IQ range as they transition to adulthood [29], from here on in this article this population of interest will be referred to by the term ASD. Structured focus groups were selected as the most appropriate data collection strategy, as the group dynamics allowed parents to construct a shared understanding of the experiences, expanding on the perspectives of individual participants, and enabling coverage of a wide range of topics in an efficient manner [30]. Structured focus group method is a variant on the traditional focus group method in that it provides the participants with information to guide the discussion after commencing with an open-ended question [30]. The group process was guided using stimulus questions that focused the discussion on transition to adulthood for young people with ASD [31]. The International Classification of Functioning, Disability and Health (ICF) [32] guided data collection and analysis, as part of a directed content analysis approach [33]. The ICF is a biopsychosocial model that conceptualises the dynamic and bi-directional interaction between health conditions, activities and contextual factors [32, 34]. Education is recognised as a basic human right [16]; in Australia equity in education has been supported by the Disability Discrimination Act (DDA) [17]. The DDA [17] has a strong focus on promotion of equity through social inclusion, and has positively impacted the education of people with disabilities[18] evidenced by the 94% increase in students with disabilities attending university since 2008 [19]. However, the DDA [17] does not mandate transition planning, a recognised gap in the current system [18], with many young people with disability still struggling to find clear pathways during their transition to adulthood.

### Participants

Nineteen parents of young people with ASD, including Asperger’s Syndrome, were recruited to participate in four structured focus groups. Recruitment was conducted using purposive and snowball sampling, via the Autism Association of Western Australia and Autism West. Participants self-selected as a “parent of a young person (aged 18–30) with ASD”. Parents required adequate English language skills to participate in a group discussion and needed to be available to attend one of the four scheduled groups. Although 21 parents initially agreed to participate in the focus groups, two parents did not attend due to other commitments.

Of the 19 parents who participated in the structured focus groups, the majority were mothers (n = 14), four were fathers and one was a step-father. Thirteen participants were partnered and they tended to have good socioeconomic status, with higher than average incomes (median = AU$80,000 –AU$100,000 per year, with a range of less than AU$20,000 to more than AU$100,000). All but two parents held university qualifications and 14 parents were in paid employment. Five of the parents were not in employment, citing their child’s ASD as the reason for their lack of workforce participation.

The parents reported on 15 male and seven female young people with ASD (Table 1), with an average age of 20.1 years (*SD* = 2.0). The young people had typically received a diagnosis on average at 4.5 years of age (*SD* = 1.0) with an average overall total score on the Social Responsiveness Scale—Second Edition (SRS-2) [35] as reported by their parents of 105.04 (SD = 25.9), with a range of 31 to 144, equating to very low to severe symptomology (S1 Table). The range of results from the SRS Restricted Interests and Repetitive Behaviour sub-scale was 6–34 (mean = 20.3; SD = 6.2) and the SRS Social Communication and Interaction was 41–114 (mean = 83.7; SD = 20.8), suggestive of a range of very mild to severe symptomology. At the time of the focus groups eight of the young people had completed a university qualification, with six yet to complete secondary schooling, ten were studying full-time and three were in paid employment. Three of the young people were unemployed, but not seeking work and a further five were seeking work and one was participating in an alternative to employment programme. The vast majority (19) of the young people were living in the family home; however, two lived in individual accommodation with support and one in shared accommodation with support.

**Table 1:** Demographic description of young adults with ASD discussed by parents in the focus groups.

Young person with ASD	Age	Gender	Employment status	Highest education	Living arrangements
1.	23	Male	Unemployed, looking for work	University education	Living in family home
2.	19	Male	Studying full-time	Post-secondary non- university education	Living in family home
3.	20	Female	Studying full-time	Post-secondary non- university education	Living in family home
4.	20	Male	Unemployed, looking for work	Post-secondary non- university education	Living in family home
5.	18	Female	Studying full-time	Secondary education	Living in family home
6.	18	Female	Studying full-time	Secondary education	Living in family home
7.	20	Male	Studying full-time	Secondary education	Living in family home
8.	20	Male	Studying full-time	Secondary education	Living in family home
9.	18	Male	Unemployed, looking for work	Secondary education	Living in family home
10.	20	Male	Unemployed, looking for work	Secondary education	Living in family home
11.	22	Female	Employed part-time (15 hours/week)	Secondary education	Living in family home
12.	20	Female	Studying full-time	Some secondary education	Living in family home
13.	20	Male	Studying full-time	Some secondary education	Living in family home
14.	18	Male	Studying full-time	Some secondary education	Living in family home
15.	18	Female	Participating in alternative employment	Some secondary education	Living in family home
16.	19	Male	Unemployed, not looking for work	Some secondary education	Living in family home
17.	21	Male	Unemployed, not looking for work	Some secondary education	Living in family home
18.	20	Male	Unemployed, looking for work	Some secondary education	Living in family home
19.	19	Male	Employed part-time (12 hours/week)	Primary education	Living in family home
20.	23	Male	Studying full-time	University education	Living in individual accommodation with support
21.	26	Male	Employed full-time	Post-secondary non-university education	Living in individual accommodation with support
22.	20	Female	Unemployed, not looking for work	Some secondary education	Living in individual accommodation without support

### Instruments

#### Demographics questionnaire

A sociodemographic questionnaire was constructed based on a similar project undertaken by the Curtin Autism Research Group [36], enabling a description of study participants.

#### Social Responsiveness Scale-2

The Social Responsiveness Scale-2 (SRS-2) [35] was employed to gauge the severity of current autistic symptoms by proxy parent report. The SRS-2 has been widely used in clinical practice and research to quantify autistic traits demonstrating high sensitivity and specificity [37–39]. A recent study of people with ASD across the lifespan utilised the SRS-2 to corroborate DSM-5 ASD diagnostic criteria with good convergence [40].

### Procedure

The structured focus groups took place at a private clinic in the Perth metropolitan region, scheduled across four weekday evenings, lasting between two to three hours. Prior to commencing each group, parents had had no prior contact with any of the researchers. Following informed consent, parents completed the sociodemographic questionnaire [36] and SRS-2 [35]. The lead author, a researcher with psychology and occupational therapy qualifications, facilitated the focus groups. The facilitator was supported by another researcher who recorded data (key statements) into a spreadsheet and took field notes. Digital voice recorders allowed for post-hoc revision of key statements.

At the commencement of each group, parents were provided with stimulus material in both written and verbal form. The stimulus question was based on contemporary views of young adulthood proposed by Blatterer [41] and posed the question, *“Thinking about your child with ASD what would help them move into adult life*?*”* Parents were encouraged to think broadly about their children’s transition to adulthood, encompassing leaving school, seeking employment, intimate relationships, learning to drive, changes in accommodation and self-determination in decisions. Prior to the focus groups, the stimulus material was piloted in a forum with 11 participants who had a range of personal and professional relationships with people with ASD. The question fostered initial open discussion between participants, with follow-up questions from the facilitator promoting interaction and discussion.

#### Data collection and in-vivo data analysis

Group discussions began with the facilitator recording keywords of the discussion on a whiteboard. Subsequently, participants added to the discussion and expanded their ideas. In collaboration with participants, meaning units raised were grouped together, with duplicates removed and units defined and condensed on a whiteboard. This strategy of expanding and then refining discussions allowed the researcher to code the condensed meaning units in-vivo with the participants. A second researcher transcribed these condensed meaning units into Microsoft Excel which were then provided to participants for member checking [42].

After a refreshment break a printed copy of the condensed meaning units was provided to each participant who were then asked to rank the three most important requirements for a person with ASD to successfully transition to adult life. Subsequently, participants were asked to rate each condensed meaning unit according to current status of the requirements for successful transition (on a 5 point scale, where 1 = poor current status and 5 = excellent current status). Participants were unaware that they would rate current status at the time they ranked the three most important condensed meaning units. The rankings and ratings were immediately entered into Microsoft Excel enabling in situ comparison and group discussion of the relative importance and status of units (see S2 Table). Participants considered whether the output was an accurate representation of the discussion and if anything had been omitted from the discussion. This process simultaneously served to identify the subcategories from focus group discussions and enable member checking, therefore increasing the trustworthiness of the data [43]. Patterns in the data of the fourth focus group supported the conclsion that saturation had been achieved, so data collection ceased [30].

#### Data analysis

Data analysis occurred in three stages: (1) in-vivo analysis during the focus groups (as described above), (2) coding findings against the ICF and (3) thematic analysis of the data. Secondary coding of the findings involved compiling the condensed meaning units from the four focus groups into codes and linking these to the ICF according to the process identified by Cieza and colleagues [44]. This involved identifying the meaningful concepts within each unit, and linking it with the most appropriate code(s). For example the code “mentoring at work and study” is associated with the condensed meaning unit “having access to supports at work”, which contains the meaningful concepts of “access”, “supports” and “work”. Hence, the ICF constructs of “interpersonal interactions and relationships, other specified (d798)”, “maintaining a job (d8451)” and “support and relationships, other specified (e398)” were selected. Systematically linking the condensed meaning units to all applicable ICF constructs [44] was completed through a process of consensus between the authors. This linking process was aided by visually representing the coded meaning units in relation to ICF constructs using Microsoft Visio.

Finally, the third stage of data analysis involved thematic analysis of the condensed meaning units grouping them into themes, associated categories and sub-categories using directed content analysis [33]. This process occurred through collaboration between researchers, and involved review of the meaning units identified during the focus groups.

### Ethical issues

This study was approved by the Curtin University Human Research Ethics Committee (HR 16/2014), written consent was obtained from all participants and all data have been managed as per the *Australian Code for the Responsible Conduct of Research* [45].

## Results

### Stage 1: Focus group in-vivo results

During the focus groups the participants raised 281 ideas relating to the potential facilitators of transition to adult life for their child with ASD (focus group median number of units = 58, range = 32–133 per group). These were distilled into 132 condensed meaning units (focus group median = 33.5, range = 28–37 per group) highlighting parents’ views of the importance of activity participation for their young adults with ASD and the impact of the environment on outcomes (see S2 Table). Across all four focus groups discussions centred on the contrast between the requirements important in a successful transition and parents’ perception of the current status. The key discrepancies related to community awareness of focusing on strengths (Focus Group 1—“*importance of people having a positive attitude towards people with ASD”*) and equal opportunities (Group 2—“*equal opportunity to embrace ASD*” and the “*need for social supports*”; Group 4 –*“work environment with social interaction”)*, along with services to facilitate success (Group 1 –“*access to information through an information centre”*). In contrast, group three overall described good current performance for the three most important condensed meaning units, which were all related to service provision (“*people who create jobs for young adults with ASD*”, “*services needed to focus on the individual*” and “*higher education providers that understand our children’s needs*”). The condensed meaning units and the summed importance rankings and average current performance ratings for each focus group are provided in S2 Table.

### Stage 2: Linkage to the ICF

The condensed meaning units identified in the four focus groups were compiled and distilled to create 22 codes (S3–S5 Tables) that were linked to the ICF, and with 45.2% of the condensed meaning units linking with the Environmental Factors domain (Table 2), 52.0% against the Participation domain (Table 3) and 2.7% against the Body Function domain (Table 4). The focus group discussions focused on the engagement in Participation in learning and applying knowledge (d1), self-care (d5), domestic life (d6), interpersonal interactions and relationships (d7), major life areas (d8), and community, social and civic life (d9). The focus group data indicated that engagement in these major life areas could be mediated by contextual factors in the Environment, including support and relationships (e3), attitudes (e4) and services, systems and policies (e5). For example, the attitudes of friends, family, peers and people in authority was described as potentially both an enabler and barrier participation. The most relevant construct in the body function domain was mental functions (b1), which was also described as a potential mediator of participation.

**Table 2:** Absolute frequencies of ICF categories from the environmental factors domain and relative frequencies across all ICF domains.

ICF code	Category code description	N (%)
e430	Individual attitudes of people in positions of authority	3 (4.1%)
e355	Health professionals	3 (4.1%)
e360	Other professionals	3 (4.1%)
e325	Acquaintances, peers, colleagues, neighbours and community members	3 (4.1%)
d815	Preschool education	2 (2.7%)
e398	Support and relationships, other specified	2 (2.7%)
e590	Labour and employment services, systems and policies	2 (2.7%)
e585	Education and training services, systems and policies	2 (2.7%)
e5902	Labour and employment policies	1 (1.3%)
e585	Education and training services, systems and policies	1 (1.3%)
d820	School education	1 (1.3%)
d825	Vocational training	1 (1.3%)
d830	Higher education	1 (1.3%)
e330	People in positions of authority	1 (1.3%)
e399	Support and relationships, unspecified	1 (1.3%)
e525	Housing services, systems and policies	1 (1.3%)
e310	Immediate family	1 (1.3%)
e315	Extended family	1 (1.3%)
e5	Services, systems and policies	1 (1.3%)
e565	Economic services, systems and policies	1 (1.3%)
e570	Social security services, systems and policies	1 (1.3%)
	Total:	33 (45.2%)

**Table 3:** Absolute frequencies of ICF categories from the participation domain and relative frequencies across all ICF domains.

ICF code	Category code description	N (%)
d825	Vocational training	4 (5.4%)
d830	Higher education	4 (5.4%)
d820	School education	3 (4.1%)
d8451	Maintaining a job	3 (4.1%)
d798	Interpersonal interactions and relationships, other specified	3 (4.1%)
d8451	Maintaining a job	2 (2.7%)
d815	Preschool education	2 (2.7%)
d6	Domestic life	2 (2.7%)
d7	Interpersonal interactions and relationships	2 (2.7%)
d810	Informal education	2 (2.7%)
d8	Major life areas	2 (2.7%)
d177	Making decisions	2 (2.7%)
d5	Self-care	1 (1.3%)
d940	Human rights	1 (1.3%)
d770	Intimate relationships	1 (1.3%)
d839	Education, other specified and	1 (1.3%)
d175	Solving problems	1 (1.3%)
d845	Acquiring, keeping and terminating a job	1 (1.3%)
d850	Remunerative employment	1 (1.3%)
	Total:	38 (52.0%)

**Table 4:** Absolute frequencies of ICF categories from the body function domain and relative frequencies across all ICF domains.

ICF code	Category code description	N (%)
b1301	Motivation	1 (1.3%)
b1648	Higher-level cognitive functions, other specified	1 (1.3%)
	Total:	2 (2.7%)

### Stage 3: Interpretation of findings

Thematic coding of meaning units from the focus groups data resulted in three main themes: ***to be understood***, ***to understand the world*** and ***to succeed***. These themes corresponded with the findings from the linkage of meaning units with the ICF, highlighting the importance of environmental aspects in transition. While the discussions within each focus group were subtly different, thematic coding revealed the data were captured within these three main themes.

#### To be understood

The theme of ***to be understood*** centred around parents’ wishes for their children ***to be understood*** by their family, peers, employers, teachers and by society in general. Parents described their wish to have the strengths of their child with ASD recognised without prejudice. Parents desired that their children be afforded equal opportunities, which they believed in part were dependent on remediating environmental barriers, see S3 Table.

The theme of ***to be understood*** is illustrated by the following quote:

*“… employers having an understanding of autism*, *that is actually the trickiest bit*,”

#### To understand the world

The parents aspired for their children ***to understand the world***, in terms of the social and institutional worlds in which they lived. Helping their children to understand the world involved preparation and support for independence and social integration are available in S4 Table. Parents referred to the mechanisms that supported their children’s understanding including the value of mentors in areas of education and work. Parents reported that mentors were at times able to act as advocates for their young person with ASD, an important role particularly given their children’s challenges with self-advocacy. Parents also expressed their hope that a mentor or other support person could help their young person with ASD understand the value of completing self-care tasks, such as teeth cleaning. While parents highly valued mentors they highlighted that they needed to be consistent and reliable, pointing to the negative impact of mentors changing or reneging on their commitments. In absence of a peer mentor, the advocacy role for the young person with ASD fell to parents, which was described as having a negative impact on the parent-child relationship.

Institutional supports were highly valued by parents, specifically those from schools including planning for transition early and building life skills through work experience and life skills training. However, parents believed that further improvements would result from more individualised tailoring of the curriculum to meet the needs of their young adults, specifically through the inclusion of more social skills and daily living training.

The theme of ***to understand the world*** can be illustrated by the following quote:

“… *someone who knew what it was like for them following them around telling them this is what you do…. Not for the job learning, but for the social aspects*…”

### To succeed

Parents expressed their desire for their young people with ASD ***to succeed*** and to reach their potential. The categories and subcategories associated with the theme ***to succeed*** are described in available in S4 Table. Parents discussed the idea that their children should be given the opportunities and supports to succeed in the areas of work and study. Parents reiterated the importance of their young person being afforded opportunities to experience success by leveraging their special interests and strengths, potentially fostering their motivation, resulting in positive experiences and building self-confidence. Parents discussed the possibility that some aspects of ASD, given the right environment and opportunities, could be harnessed and help their children to succeed.

Living arrangements were discussed at length by parents, but their definitions of ‘successful’ living arrangements were highly individualised. Many preferred semi-detached living arrangements, such as a “granny flat” or a self-contained studio within the family home, preferences linked with the realisation that their young person was likely to need ongoing support in their daily lives. Others described their aspirations for their child to live independently in their own homes, see S5 Table.

The theme of ***to succeed*** can be illustrated by the following quote:

“… *they need self-belief, self-efficacy. They are so often told what they can’t do and when they get some positive feedback they feel great*.”

#### Relationships with the ICF

In relation to the ICF the theme ***to be understood*** contributes to several areas. The majority of subcategories within this theme mapped against the domain of the e*nvironment*. However, it also links to constructs within *body functions* and *personal factors*. The theme ***to understand the world*** can be mapped predominantly to the ICF domain of the e*nvironment*. However, it also links to the ICF domains of *body function* and *personal factors*. ***To succeed*** links to several domains of the ICF, with the majority of the subcategories in this theme linking to constructs within the e*nvironment* domain. It also mapped to constructs within the domains of *body functions* and *personal factors*. S3–S5 Tables outline the themes, categories and subcategories mapped according to the ICF.

## Discussion

Young people with ASD predominantly live in the family home and parents continue to play a vital role in the transition to adulthood [9, 46, 47], giving credence to their perspective in understanding the transition to adulthood of young people with ASD within the normative IQ range. The perspectives of parents and adolescents’ perceptions may diverge during this period, and future research should explore the views of young adults with ASD. However, it is unlikely that focus groups or interviews enable optimal qualitative data collection in this group given their communication difficulties, as such alternative techniques such as Q-methodology should be considered [48–50]. While representative sampling and generalisation is not the goal of qualitative research, findings from this study are potentially transferable [51], as they provide an in-depth description of the perspective or parents of young adults with ASD in the normative IQ range. The authors identified three themes from the data: ***to be understood***, to ***understand the world*** and ***to succeed***. These themes have the potential to influence service design and provision for young people and parents alike.

### To be understood

The theme ***to be understood*** highlights the belief of parents that young people with ASD are marginalised because of their diagnostic characteristics, and particularly because of their social deficits [52]. The parents suggested that this was especially problematic given that ASD does not have any physical markers, and in the absence of an intellectual disability, there was an assumption that their children required little or no assistance. The social deficits of ASD continue to have implications for an individual even if they have an average or above average IQ [53], and they require understanding from employers [54], education providers, and service providers to facilitate their full participation in society.

***To be understood*** encompassed participants’ belief that aspects of ASD could be harnessed in a strengths-based approach, suggesting a move beyond equal opportunity to approaches which recognise the specialised skills and abilities of people with ASD. This shift in paradigm to a strengths-based approach, rather than the historical deficit viewpoint, was reiterated across the focus groups [55]. Data analysis revealed that this idea was also linked with the importance of person-centred approaches in improving outcomes for young people with ASD [56].

***To be understood*** highlighted the importance of maximising the person-environment fit of these young people. Flexible work practices tailored to the needs of the individual with ASD can facilitate workplace success and fit [50]. Maximising this fit includes ensuring employers and colleagues are knowledgeable of the characteristics of people with ASD, who may require accommodations such as minimising external stimuli and a workplace that is predictable and supportive [50]. The role that being understood plays in facilitating workplace success for people with ASD [50, 57, 58] is evident in the finding that while employees with ASD still require support in ASD specific workplaces it is substantially less than in open employment [57]. A holistic understanding of people with ASD should encompass the notion that many possess strengths and abilities that are advantageous in the workplace and that can be utilised in society [58, 59]. Fundamental to this understanding is recognition of individuality of people with ASD [55, 60], and the role of tailored services and supports in meeting their needs and capitalising on their strengths. While participants in this study recognised that a person-centred approach is already evident in some services [56], there is significant room for improvement.

### To understand the world

The parents also recognised the bidirectional relationship between their young person with ASD and society, recognising the need for their young person with ASD ***to understand the world***. This theme highlighted the challenges that young people with ASD experience with some aspects of social interaction, such as emotion recognition and symbolic communication [59]. While there has been hope that these difficulties would be in part be remediated by early intervention [61], the experience of the parents in these focus groups suggest that there is a need for life-long treatment options [62]. Peer mentoring is one possible life-long approach that provides an opportunity for a young person with ASD to learn in vivo about their environment, avoiding the need to translate skills from training contexts to real-life [63]. Peer mentoring could potentially reduce the requirement for parents’ to support their young person with ASD in some situations, positively impacting on the relationship between parents and their young person with ASD [64].

### To succeed

The final theme ***to succeed*** was characterised by parents expressing their desire for their young person to become a successful adult, however, parents’ definitions of success varied greatly. For an individual with ASD success as an adult is facilitated by finding their niche [65] and building self-efficacy through performance mastery [58, 66]. Matching the strengths and skills of adults with ASD to employment environments and tasks fosters success [57] and reduces the need for support [57], contributing to a positive employment relationship likely to perpetuate success in other life areas [50, 67].

### The use of the ICF

This study demonstrated the utility of the ICF in research, providing a framework which facilitated data analysis. By elucidating the areas of greatest potential need, the ICF provides important information to researchers and clinicians on the directions for future research and intervention targets [55]. The use of the universal taxonomy of the ICF enables the data to be interpreted and utilised by various professionals and sectors. Overall, the findings from this research highlight the environment as a potential mediating factor in supporting young people with ASD in their transition to adulthood and their participation in major life areas. The holistic examination of ASD using the ICF in this study and in other projects such as the ASD core sets (53, 58, 66, 67), highlight the strengths of people with ASD. Individualised and strengths-based approaches that utilise environmental adaptation could ultimately, improve outcomes for adults with ASD (58).

The findings of this study should be interpreted with the following limitations in mind. The majority of participants were members of various Autism associations, which may have shaped their experiences. Participants were from relatively high socioeconomic backgrounds and likely to have had access to significant financial resources. Their experiences may not represent those with fewer economic resources. It should also be noted that this study did not describe the experiences of parents of people with ASD who had an intellectual disability, but rather provided an in-depth description of those in the normative range. The results also need to be interpreted with some caution as the study did not explore the viewpoints of young people with ASD. While the study sample was small, purposive sampling enabled a rich exploration of the phenomenon serving to enhance the trustworthiness of the data, specifically the transferability of the findings [68].

There is a paucity of evidence addressing the transition to adulthood for young people with ASD. Findings from this research highlight that the environment as an important factor in influencing transition outcomes. Future research should examine how the environment can be modified to facilitate success and how individuals can be guided to seek out a future which meets their aspirations. It would also be of interest to further examine how gaining employment for people with ASD can be fostered and maintained. While this study provided an in-depth description of the viewpoints of parents of young adults with ASD, a similar study should be undertaken with young people with ASD themselves and parents of youth with ASD and an intellectual disability.

## Conclusion

The findings from these focus groups highlight the potential of modifying the environment as an approach in working with young people with ASD [69, 70]. Such environmental adaptations could augment other interventions for young people with ASD and ultimately serve to support their participation in major life areas. Transition to adulthood represents a critical period for young people with ASD and their families, a time when supports are needed to maximise the opportunities for young people with ASD to achieve their ambitions and participate fully in society [71].

## Supporting information

S1 Table Clinical description using the SRS-2 and age of diagnosis of young adults discussed in the focus groups. (DOCX)

S2 Table Focus group participant’s responses to the stimulus question “Thinking about your child with ASD what would help them move into adult life?”. (DOCX)

S3 Table The distilled quotes, condensed meaning units, codes, sub-categories and categories within the theme of *to be understood*. (DOCX)

S4 Table The distilled quotes, condensed meaning units, codes, sub-categories and categories within the theme of *to understand the world*. (DOCX)

S5 Table The distilled quotes, condensed meaning units, codes, sub-categories and categories within the theme of *to succeed*. (DOCX)
