# Supplementary material for: To be understood: Transitioning to adult life for people with Autism Spectrum Disorder
Source: PLoS One. 2018 Mar 26;13(3):e0194758. doi: 10.1371/journal.pone.0194758 (PMC5868819; doi:10.1371/journal.pone.0194758)
Supplement: S1 Table — (DOCX) [file pone.0194758.s001.docx]

S1 Table. Clinical description using the SRS-2 and age of diagnosis of young adults discussed in the focus groups.

|  |  | **Social Responsiveness Scale (SRS) - Second Edition** | | |
| --- | --- | --- | --- | --- |
| **Young person with ASD** | **Age of diagnosis** | **SRS Restricted Interests and Repetitive Behaviour** | **SRS Social Communication and Interaction** | **SRS total raw score** |
|  | 2 | 13 | 83 | 96 |
|  | 2 | 12 | 68 | 80 |
|  | 5 | 23 | 105 | 128 |
|  | 5 | 28 | 113 | 141 |
|  | 4 | 22 | 75 | 97 |
|  | 4 | 23 | 105 | 128 |
|  | 6 | 6 | 31 | 37 |
|  | 6 | 22 | 95 | 117 |
|  | 4 | 20 | 94 | 114 |
|  | 4 | 24 | 90 | 114 |
|  | 6 | 22 | 74 | 96 |
|  | 5 | 14 | 75 | 89 |
|  | 5 | 18 | 84 | 102 |
|  | 4 | 13 | 41 | 54 |
|  | 5 | 25 | 114 | 139 |
|  | 4 | 28 | 86 | 114 |
|  | 4 | 20 | 73 | 93 |
|  | 4 | 20 | 87 | 107 |
|  | 4 | 34 | 110 | 144 |
|  | 5 | 21 | 91 | 112 |
|  | 6 | 21 | 75 | 96 |
|  | 5 | 17 | 73 | 90 |
| Range | 2-6 | 6-34 | 41-114 | 37-144 |
| Mean (SD) | 4.5 | 20.3 (6.2) | 83.7 (20.8) | 104.0 (26.0) |
