# Supplementary material for: To be understood: Transitioning to adult life for people with Autism Spectrum Disorder
Source: PLoS One. 2018 Mar 26;13(3):e0194758. doi: 10.1371/journal.pone.0194758 (PMC5868819; doi:10.1371/journal.pone.0194758)
Supplement: S2 Table — (DOCX) [file pone.0194758.s002.docx]

**S2 Table. Focus group participant’s responses to the stimulus question “Thinking about your child with ASD what would help them move into adult life?”**

| **Focus group 1 (n=6)** | | | **Focus group 2 (n=4)** | | | **Focus group 3 (n=4)** | | | **Focus group 4 (n=5)** | | |
| --- | --- | --- | --- | --- | --- | --- | --- | --- | --- | --- | --- |
| Condensed meaning unit^a^ | **Total importance^b^** | **Average performance^c^** | **Condensed meaning unit ^a^** | **Total importance^b^** | **Average performance^c^** | **Condensed meaning unit^a^** | **Total importance^b^** | **Average performance^c^** | **Condensed meaning unit^a^** | **Total importance^b^** | **Average performance^c^** |
| Mentors in school | 2.0 | 1.2 | Future planning |  | 2.3 | Work experience |  | 0.0 | ‘Granny flat’ living at the family home | 3.0 | 2.4 |
| Mentors in life | 1.0 | 1.5 | Information about services | 1.0 | 1.2 | Supported employment services |  | 2.2 | Supported living budget |  | 2.6 |
| Mentors in health | 1.0 | 1.7 | Family network |  | 3.0 | Matching strengths and capabilities |  | 3.25 | Advocate | 1.0 | 2.6 |
| Understanding in the community | 1.0 | 1.2 | Family belief and trust |  | 2.5 | Using personal contacts and networks | 4.0 | 1.5 | Self-advocacy |  | 2.0 |
| Understanding in the near family | 2.0 | 2.7 | Acceptance of differences | 1.0 | 0.8 | Life skills training |  | 2.5 | Developing self-confidence | 2.0 | 2.6 |
| Disclosure of ASD | 1.0 | 2.8 | Social support systems | 6.0 | 1.7 | Social skills training |  | 3.5 | Understanding employer |  | 2.6 |
| Apprenticeships | 1.0 | 1.5 | Understanding from the employers |  | 1.2 | Specialist school environments |  | 2.5 | Mentors in life | 3.0 | 3.0 |
| Forward planning | 1.0 | 2.0 | Understanding from educators |  | 1.0 | Mainstream school environments |  | 3.2 | Developing independence |  | 2.2 |
| Ongoing planning | 1.0 | 1.8 | Adaptive job recruitment |  | 1.2 | Access to funding |  | 2.2 | Opportunity to make own mistakes |  | 2.6 |
| Education options | 1.0 | 1.7 | Matching interest to occupation |  | 1.7 | Access to information | 2.0 | 2.8 | Gradual independence | 3.0 | 2.8 |
| Tailored Government support | 1.0 | 1.3 | ASD adapted recruitment processes |  | 1.2 | Consistency is important |  | 3.0 | Foster daily living skills |  | 2.6 |
| Gentle staged transition | 2.0 | 1.5 | Focus on work performance |  | 1.2 | Services need to focus on the individual |  | 2.2 | Transition planning early |  | 3.0 |
| Information centre | 1.0 | 1.7 | Social stories for employees |  | 1.3 | Supported transition programs | 6.0 | 3.0 | Individualised services/strategies |  | 2.4 |
| Better knowledge in the health service | 1.0 | 1.5 | Recognizing contribution from employees with ASD | 2.0 | 1.2 | Higher education providers that understand ASD |  | 2.2 | Sex education, especially for girls |  | 2.4 |
| ‘Buddy’ mentoring | 1.0 | 2.0 | Non-traditional job framing |  | 1.3 | Being proactive | 3.0 | 3.2 | Ownership of ASD |  | 2.6 |
| Respite | 2.0 | 1.7 | Bosses with ‘no ego’ |  | 0.8 | Planning for adult life early |  | 4.5 | Disclosure of ASD |  | 2.8 |
| Family support | 1.0 | 2.2 | Employment agencies supporting people with ASD |  | 2.0 | Inclusive planning processes | 1.0 | 3.0 | Understanding employers |  | 2.2 |
| Employment co-ordinator | 1.0 | 1.8 | Support person for job retention |  | 1.7 | Knowing other parents |  | 1.8 | Understanding from education providers |  | 2.6 |
| School involvement in the transition | 1.0 | 1.0 | Mentoring study |  | 1.8 | Having access to supports at school |  | 3.2 | Strategies to encourage emotional regulation |  | 3.4 |
| Role model with ASD | 1.0 | 1.0 | Mentoring work | 1.0 | 1.3 | Having access to supports at university |  | 3.5 | Contact point schools, employers |  | 2.6 |
| Mentor at work | 1.0 | 1.5 | Employment agencies supporting people with ASD |  | 1.5 | Having access to supports at work |  | 2.5 | Consistent services |  | 2.0 |
| Supervisors need to know what the problems are | 1.0 | 1.3 | ‘Peer-like’ mentor |  | 2.2 | Information about adult services |  | 3.2 | Ongoing support/services | 3.0 | 2.2 |
| Be treated with respect | 1.0 | 1.8 | Not being singled out from others |  | 1.2 | Tapping into their interests | 2.0 | 3.0 | Provide hope |  | 3.0 |
| Government employing people with disabilities | 1.0 | 1.2 | ‘Equal opportunity’ to embrace ASD | 7.0 | 1.0 | Employers having and understanding of Autism |  | 3.0 | Assistance to find work | 1.0 | 2.6 |
| Employers aware of potential of people with ASD | 1.0 | 1.3 | Targeted curriculum for ASD |  | 1.2 | Opportunities for open employment |  | 2.8 | Support in the workplace |  | 3.0 |
| Matching jobs with interests and skills | 1.0 | 1.2 | Matching interest to education |  | 2.0 | Consistency in service providers is important |  | 3.5 | Finding a niche to foster confidence | 1.0 | 2.8 |
| Positive attitudes towards people with ASD | 1.0 | 1.3 | Lifelong support for the person with ASD | 3.0 | 1.8 | Sharing the transition plan |  | 3.2 | Teaching life skills | 3.0 | 3.0 |
| Training for independence | 1.0 | 1.3 | Housing transition ‘duplex concept’ |  | 2.0 | Jobs for young adults with ASD |  | 3.0 | Support and supervision in employment | 2.0 | 2.6 |
| Option to live independently | 1.0 | 1.5 | Sensitivity to vulnerability from peers |  | 1.2 |  |  |  | Open to ideas for training – using abilities |  | 2.6 |
| Meeting housing/accommodation needs | 1.0 | 1.5 | Stepwise independence program | 3.0 | 2.2 |  |  |  | A social work environment | 6.0 | 2.8 |
| Support applying daily living skills | 1.0 | 1.3 | Identify motivational drives |  | 2.7 |  |  |  | Mentors and services |  | 2.2 |
| ‘Granny flat solution’ | 1.0 | 1.5 | Facilitating decision making |  | 2.0 |  |  |  | A 'friend' or buddy system | 2.0 | 2.6 |
| Personal assistant explaining to others | 1.0 | 1.3 | Find social groups through the internet |  | 2.0 |  |  |  |  |  |  |
| Full time support Government recognition | 1.0 | 1.3 | Intimate relationship information |  | 1.2 |  |  |  |  |  |  |
| Centre for young adults | 1.0 | 1.2 | Financial implications |  | 0.0 |  |  |  |  |  |  |
| Respite | 2.0 | 1.7 |  |  |  |  |  |  |  |  |  |
| Support also for those outside school | 1.0 | 1.3 |  |  |  |  |  |  |  |  |  |
| *Note:*  ^a^ Responses to the stimulus question were analysed in-vivo into condensed meaning units.  ^b^ Participants in each focus group were instructed to rank the three most important (3=most important) condensed meaning units or requirement for a person with ASD to transition to adult life, therefore higher ranks equate with greater importance.  ^c^ Participants were asked to rate each condensed meaning unit or requirement according to current performance (on a 5 point scale, where 1=poor performance and 5=excellent performance) and this was averaged to ascertain the current performance. | | | | | | | | | | | |
