# Supplementary material for: To be understood: Transitioning to adult life for people with Autism Spectrum Disorder
Source: PLoS One. 2018 Mar 26;13(3):e0194758. doi: 10.1371/journal.pone.0194758 (PMC5868819; doi:10.1371/journal.pone.0194758)
Supplement: S3 Table — (DOCX) [file pone.0194758.s003.docx]

S3 Table. The distilled quotes, condensed meaning units, codes, sub-categories and categories within the theme of *To Be Understood.*

| **Theme 1: To Be Understood** | | | | |  |
| --- | --- | --- | --- | --- | --- |
| **Illustrative quotes** | **Condensed Meaning Unit**  **(Focus Group Number)** | **Code**  **ICF Constructs** | **Sub-category**  **ICF Single Level Constructs** | **Category** | |
| *He wants to – know he can be normal. Because he is not in school. So in high school it is difficult. They don’t want to be singled out.* | - Not being singled out from others (2) - Sensitivity to vulnerability from peers (2) - Acceptance of difference (2) | **Not being singled out by others**   - Individual attitudes of: - Friends (e420) - Acquaintances, peers, colleagues, neighbours and community members (e425) | - Attitudes (e4) | Inclusive communities | |
| *We are on the verge of something big, in the same way that equal opportunity and affirmative action and – of which they have made use of affirmative action to meet some targets. But – equal opportunity, and all the things that have surrounded that over the last 50 or 60 years or whatever – what year is it? 2014?* | - Understanding in the community (1) - Understanding in the near family (1) - Disclosure of autism (1) - Equal opportunity' to embrace ASD (2) - Understanding from educators (2) - Understanding from education providers (3) - Disclosure of ASD (3) - Ownership of ASD (3) | **Equal opportunities to embrace ASD**   - Human rights (d940) - Individual attitudes of: - Friends (e420) - Acquaintances, peers, colleagues, neighbours and community members (e425) - People in positions of authority (e430) | - Community, social and civic life (d9) - Attitudes (e4) |  |  |
| *He’s been linked up to a disability employment provider… I mean he’s in his second year there now, and [they] still come in because it’s not just the getting of the job, it’s also the maintenance over time* | - Family support (1) - Personal assistant explain to others (1) - Social support systems (2) - Find social groups through the Internet (2) - Social stories for employees (2) - Family network (2) - Family belief and trust (2) - Work environment with social interaction, not working alone and isolated (3) - Someone to advocate for person with ASD (3) - Self-advocacy (3) - Use personal contacts / networks (4) | **Social support systems**   - General social support services (e5750) | - Services, systems and policies (e5) |  |  |
| *There have been mechanisms for accommodating people. Hopefully that is not being lost. I fear perhaps the university system has been driven to a profit-centred frame where even accommodating people with what might have been traditionally found some solace, can’t.* | - Having higher education providers that understand our children’s needs (3) | **Understanding from educators**   - School education (d820) - Vocational training (d825) - Higher education (d830) - Individual attitudes of people in positions of authority (e430) | - Major life areas (d8) - Attitudes (e4) | Supportive study and work environments | |
| *There’s a lot of bosses out there that have got egos so there’s a lot of bosses that feel like if somebody is “weird”, it is the same as school. They don’t want you. So my brother’s boss is just an example – he’s just a genuine, great guy. Who doesn’t have an ego. He’s just noticing the valuable – you know.* | - Be treated with respect (1) - Bosses with no ego (2) - Employer to support self-belief (3) | **Bosses with no egos**   - Individual attitudes of people in positions of authority (e430) | - Attitudes (e4) |  |  |
| *His boss is amazing. He recognises this and he’s got an approach to managing him.* | - Supervisors need to know what the problems are (1) - Focus on work performance (2) - Acceptance of difference (2) - Understanding from the employers (2) - Understanding employers (3) - Employers have an understanding of Autism (4) | **Focus on work performance**   - Maintaining a job (d8451) - Individual attitudes of people in positions of authority (e430) | - Major life areas (d8) - Attitudes (e4) |  |  |
| *He’s in a job now where the – his boss actually recognises that this guy’s got some incredible memory skills.* | - Employer realise the potential of people with autism (1) - Positive attitudes towards people with autism (1) - Recognising special contribution from employees with ASD (2) - Better knowledge in the health service (4) | **Recognition of special contribution of employees with ASD**   - Maintaining a job (d8451) - Labour and employment policies (e5902) | - Major life areas (d8) - Services, systems and policies (e5) | Strengths focus | |
