# Supplementary material for: To be understood: Transitioning to adult life for people with Autism Spectrum Disorder
Source: PLoS One. 2018 Mar 26;13(3):e0194758. doi: 10.1371/journal.pone.0194758 (PMC5868819; doi:10.1371/journal.pone.0194758)
Supplement: S4 Table — (DOCX) [file pone.0194758.s004.docx]

S4 Table. The distilled quotes, condensed meaning units, codes, sub-categories and categories within the theme of *To Understand the World.*

| **Theme 2: To Understand the World** | | | | |
| --- | --- | --- | --- | --- |
| **Illustrative quotes** | **Condensed Meaning Unit**  **(Focus Group Number)** | **Code**  **ICF Constructs** | **Sub-category**  **ICF Single Level Constructs** | **Category** |
| *So at school, if they need to be educated, and they really want an education, and they really want to learn – but the school environment, and the education system – just chews our kids up and spits them out, and I don’t think very many of them actually succeed and get through.* | - Education options (1) - Targeted curriculum for ASD (2) | **Targeted ASD curriculum**   - Preschool education (d815) - School education (d820) - Vocational training (d825) - Higher education (d830) - Education and training services, systems and policies (e585) | - Major life areas (d8) Services, systems and policies (e5) | Preparation for independence and social integration |
| *We were just so impressed because of the life skill training that they [the employment agency] did with him catching buses, and taking cash from home and our accounts in order to you know, pay them at post offices and that sort of thing. Learning to catch public transport, over and over again, different routes around Perth. How to look up timetables, and I think that is all been very valuable for him in terms of moving into adult life, and we now work with an agency called “Interchange” and on Wednesdays they specifically still keep looking at life skills, in terms of even cooking and cleaning at home.* | - Training for independence (1) - Support applying daily living skills (1) - Social Skills training/Structured social groups (1) - Intimate relationship information (2) - Teaching life skills and start early (3) - Foster practical skills - for the day to day (3) - Try for adult children to be independent in dressing, paying bills, cleaning, washing sheets, changing clothes (3) - Teaching strategies to deal with emotional and encourage self and emotional regulation (3) - Sexual education. Need to protect our young adults, especially girls (3) - Life skills training (4) | **Skills for adult life**   - Self-care (d5) - Domestic life (d6) - Interpersonal interactions and relationships (d7) - Intimate relationships (d770) - Informal education (d810) | - Self-care (d5) - Domestic life (d6) - Interpersonal interactions and relationships (d7) - Major life areas (d8) |  |
| *I’ve always wanted to get – have a mentor for my son… You know, had the same interests… Yeah, someone who can say ‘I’ve been there, done that’.* | - Role model with autism (1) | **Social stories for employees with ASD**   - Interpersonal interactions and relationships (d7) - Informal education (d810) - Maintaining a job (d8451) | - Interpersonal interactions and relationships (d7) - Major life areas (d8) |  |
| *You are doing it right now without help so it won’t work, but it is important to have other people coming in and if – and if you imagine that it should be recognised as needing lifelong support that you need for a boy with autism, even if he is an adult* | - Lifelong support for person with ASD (2) - Ongoing support / services (3) - Communication is important for schools, employers. need to have a contact point (3) | **Lifelong support for the person with ASD**   - Acquaintances, peers, colleagues, neighbours and community members (e325) - People in positions of authority (e330) - Health professionals (e355) - Other professionals (e360) - Support and relationships, other specified (e398) | - Support and relationships (e3) | Supporting independence and social integration |
| *I’ve always wanted a mentor for my son, someone to help them feel confident.* | - Support also for those outside school (1) - Mentor at work (1) - Mentoring study (2) - Mentoring work (2) - Mentor in school (3) - Mentor in life (3) - Mentor in health (3) - Services / mentorship must be available (3) - Mentors at university, school, work (4) - Access to supports at school (4) - Access to supports at university (4) - Having access to supports at work (4) | **Mentoring at work or study**   - Interpersonal interactions and relationships, other specified (d798) - Vocational training (d825) - Higher education (d830) - Education, other specified and unspecified (d839) - Maintaining a job (d8451) - Acquaintances, peers, colleagues, neighbours and community members (e325) - Support and relationships, other specified (e398) | - Interpersonal interactions and relationships (d7) - Major life areas (d8) - Support and relationships (e3) |  |
| *I wish there was a buddy, someone to go for a coffee with… that would build him up* | - "Buddy" mentoring (1) - Peer like - cool mentor (2) - People of their own age, having a 'friend' or buddy system (3) | **Peer like - cool mentor**   - Interpersonal interactions and relationships, other specified (d798) - Acquaintances, peers, colleagues, neighbours and community members (e325) | - Interpersonal interactions and relationships (d7) - Support and relationships (e3) |  |
| *The school based apprenticeship with a support of an employment coordinator was working really well, it was a really gentle transition. She had the social trainers from school and an employment coordinator that checked on her.* | - Employment co-ordinator (1) - Employment agencies supporting people with ASD (2) - Support person for job retention (2) - Must be supported and supervised in the employment setting (3) - Support in the workplace (3) | **Support for job retention**   - Interpersonal interactions and relationships, other specified (d798) - Maintaining a job (d8451) - Health professionals (e355) - Other professionals (e360) - Labour and employment services, systems and policies (e590) | - Major life areas (d8) - Support and relationships (e3) - Services, systems and policies (e5) |  |
