# Supplementary material for: To be understood: Transitioning to adult life for people with Autism Spectrum Disorder
Source: PLoS One. 2018 Mar 26;13(3):e0194758. doi: 10.1371/journal.pone.0194758 (PMC5868819; doi:10.1371/journal.pone.0194758)
Supplement: S5 Table — (DOCX) [file pone.0194758.s005.docx]

S5 Table. The distilled quotes, condensed meaning units, codes, sub-categories and categories within the theme of *To Succeed.*

| **Theme 3: To Succeed** | | | | | |
| --- | --- | --- | --- | --- | --- |
| **Illustrative quotes** | **Condensed Meaning Unit**  **(Focus Group Number)** | **Code**  **ICF Constructs** | **Sub-category**  **ICF Single Level Constructs** | **Category** |  |
| *You know, her narrow interests. Very specialised, very good, very good – you know. It would be great if someone paid her to do all that.* | - Job matching with interest and skills (1) - Matching interest to occupation (2) - Matching interest to education (2) - Identify motivational drives (2) - Open to ideas for training - using abilities (3) - Finding their niche and using it to foster involvement + confidence (3) - Focus and matching strengths and capabilities to environments (4) - Tapping into their interests (4) | **Identifying motivational drives**   - Motivation (b1301) - Higher-level cognitive functions, other specified (b1648) - Major life areas (d8) - Preschool education (d815) - School education (d820) - Vocational training (d825) - Higher education (d830) | - Mental functions (b1) Major life areas (d8) - Services, systems and policies (e5) - Learning and applying knowledge (d1) - Support and relationships (e3) - Major life areas (d8) Services, systems and policies (e5) - Domestic life (d6) - Support and relationships (e3) - Major life areas (d8) Support and relationships (e3) - Services, systems and policies (e5) - Services, systems and policies (e5) - Services, systems and policies (e5) | Planning for success |  |
| *Schools need to get more involved in the students transition* | - Forward planning (1) - Ongoing planning (1) - Future planning (2) - Provide hope (3) - Supported living - having a budget (3) - Being proactive is important (4) - Inclusive planning processes which included people outside of the disability sector (4) - Planning for adult life early (4) | **Planning for adult life**   - Solving problems (d175) - Making decisions (d177) - Education and training services, systems and policies (e585) |  |  |  |
| *I certainly need to think differently about how she can make her own decisions and come to her own conclusions, and perhaps facilitate it rather than direct. So facilitating is as – there’s a temptation to jump straight to this direction.* | - Facilitating decision making (2) | **Facilitating decision making**   - Making decisions (d177) - Support and relationships, unspecified (e399) |  |  |  |
| *The school based apprenticeship with a support of an employment coordinator was working really well, it was a really gentle transition. She had the social trainers from school and an employment coordinator that checked on her.* | - Gentle staged transition (1) - School involvement in the transition (1) - Specialist School Environments (1) - Stepwise independence program (2) - Start the transition as early as possible (3) - Let them make their own mistakes up to some point - it is part of learning. To their own level and steady increments (3) - Gradual independence. Foster practical skills - For the day to day (3) - Self-confidence - giving him the confidence to do it again (3) - Everyone involved knowing the transition plan (4) - Mainstream School Environments (4) - Supported transition programs that gradually withdraw (4) | **Stepwise independences programs**   - Major life areas (d8) - Education and training services, systems and policies (e585) |  | Gradual progress towards success |  |
| *A couple of years ago, I thought the best thing we could do maybe is have [build] a duplex.* | - Option to live independently (1) - Matching housing/accommodation to needs (1) - ‘Granny flat solution’(1) - Housing transition 'duplex concept' (2) - Granny flat living - cottage on the side. Adult children are living at home but they should live alone (3) | **Housing transitions**   - Domestic life (d6)   Housing services, systems and policies (e525)   - Immediate family (e310) - Extended family (e315) |  |  |  |
| *I would say, you know, you have got to have roles that can accommodate people, and as a process of selection, that doesn’t rely on traditional mechanisms, because selecting someone based on interview – you are not selecting them because they are good at interviewing, you are selecting them because they are good at whatever the job is.* | - Government decision to employ people with disabilities (1) - Supported employment services (1) - Opportunities for open employment (1) - Centre for young adults (1) - Assistance to find work (3) - Adaptive job recruitment (3) - ASD adapted recruitment processes (3) - Untraditional framing of job (3) - Apprenticeship (3) - Work / practical experience (4) - Having people who will create jobs for young adults with Autism (4) | **Specialised employment services**   - Acquiring, keeping and terminating a job (d845) - Remunerative employment (d850) - Health professionals (e355) - Other professionals (e360) - Labour and employment services, systems and policies (e590) |  |  |  |
| *I am learning all the time as to what is available and what isn’t available, because it is – it is very changeable, and – you know, it is good to have current information.* | - Information centre (1) - Information about service (2) - Opportunities to gain knowledge about services for adult life (4) - Access to information (4) | **Access to information**   - Services, systems and policies (e5) |  | Services to maintain success |  |
| *[Services] meeting the individual needs of the person.*  *There was no funding, and I – with an Asperger’s diagnosis* | - Tailored government support (1) - Full time support - government recognition (1) - Respite (3) - Consistency is important (3) - Consistency in service providers is important (3) - Consistent Services (3) - Services need to focus on the individual (3) - Tailor services/strategies to the person (3) - Access to Funding (4) - Knowing other parents (4) | **Financial implications**   - Economic services, systems and policies (e565) - Social security services, systems and policies (e570) |  |  |  |
